# A 10-year study of *Alternaria* and *Cladosporium* in two Polish cities (Szczecin and Cracow) and relationship with the meteorological parameters

**DOI:** 10.1007/s10453-015-9411-5

**Published:** 2015-11-14

**Authors:** Agnieszka Grinn-Gofroń, Agnieszka Strzelczak, Danuta Stępalska, Dorota Myszkowska

**Affiliations:** Department of Plant Taxonomy and Phytogeography, University of Szczecin, Wąska 13, 71-415 Szczecin, Poland; Department of Process Engineering, West Pomeranian University of Technology, Szczecin, Poland; Institute of Botany, Jagiellonian University, Cracow, Poland; Department of Clinical and Environmental Allergology, Jagiellonian University Medical College, Cracow, Poland

**Keywords:** Temporal trend aerobiological monitoring, Meteorological parameters, Fungi spores, Spore season, Ordination method

## Abstract

*Alternaria* and *Cladosporium* spores belong to the most frequent and allergenic particles in bioaerosol in the temperate climate. The investigation of *Alternaria* and *Cladosporium* spore concentrations was performed in two cities in Poland, Szczecin and Cracow, in 2004–2013. The meteorological parameters taken to assess their impact on fungal spores were average, maximum and minimum temperature, relative humidity and average wind velocity. In order to reveal whether changes in dynamics of spore seasons are driven by meteorological conditions, ordination methods were applied. Canonical correspondence analysis was used to explore redundancy among the predictors (meteorological parameters). Prior to ordination analyses, the data were log(*x*)-transformed. Concentrations of *Alternaria* and *Cladosporium* spores were significantly higher in Szczecin comparing to Cracow, but it was also observed the decreasing trend in the spore concentrations in Szczecin. As regards temperature, it was higher in Cracow and was still increasing in the studied years. Relative humidity and wind velocity were significantly lower in Cracow. In Szczecin meteorological conditions did not explain changes in spore season characteristics (insignificant redundancy analysis models), while in Cracow’s redundancy analysis models indicated that spore season parameters were in over 40 % determined by meteorological conditions, mainly air temperature and wind velocity. If they increase, the peak value, total number of spores and their average concentrations in a season will also increase.

## Introduction

Fungal spores constitute a significant component of bioaerosol and can be found in the air at almost any time throughout the year. Most fungal species act saprotrophically, colonizing all stages of plant growth, or as plant pathogens, prompting considerable economic losses worldwide (Hjelmroos 1993; Infante et al. 1999a, b). *Cladosporium* and *Alternaria* are the most cosmopolitan fungi, particularly in temperate regions, due to their almost permanent presence outdoor and indoor (Infante et al. 1999a, b; D’Amato 1981; Ricci et al. 1995).

*Cladosporium* is the most abundant spore type in many outdoor environments, accounting for 40–80 % of the total spore count in many European cities (Spieksma 1995), while *Alternaria* is included in many studies because of its importance in the agricultural sector due to its pathogenicity towards different crops such as potatoes (Iglesias et al. 2007). Both spore types are of clinical importance because they are considered to be allergenic (Kurup et al. 2002). The monitoring of fungal spores in Poland revealed the summer as the most favourable season for *Alternaria* and *Cladosporium* occurrence (Stępalska et al. 1999; Konopińska 2004; Grinn-Gofroń and Strzelczak 2008a, b).

In the present study, we investigate the daily *Alternaria* and *Cladosporium* concentrations in two cities of Poland—Szczecin and Cracow. The main goals of the research were to: (1) broaden the global knowledge on airborne spore dynamics based on the first Polish long-term data for a multiannual period (2004–2013); (2) reveal long-term trends of changes in spore season characteristics, (3) assess the driving factors of those changes in relation to meteorological conditions.

## Materials and methods

**Fig. 1:**
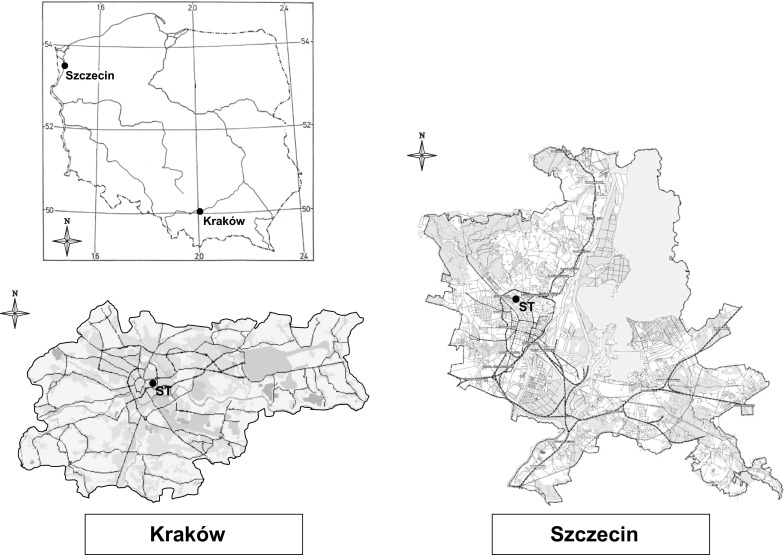
Location of sampling sites

The investigation of the *Alternaria* and *Cladosporium* daily spore concentrations in the atmosphere was carried out in two cities in Poland, Szczecin and Cracow, in 2004–2013 (Fig. 1). Both cities are surrounded by forests, farmlands and abandoned farmland areas which provide suitable media for the spore production. The maritime climate of Szczecin is influenced by impact of the air masses from over the Northern Atlantic. Most often the polar air masses are characterized by high humidity, which affects the growth of the summer cloud cover and amount of precipitation. Winter is associated with warming and high cloudiness. These masses are most common in summer and autumn. The mean annual temperature is 8.6 °C and the annual precipitation—550 mm. The average wind speed is 3.3 m s^−1^ and western (W) and south-western (SW) wind directions are dominant (Woś 1999).

Cracow is situated in a region where weather changes are frequent due to the friction of both humid air masses arriving from over the Northern Atlantic Ocean and dry, continental masses of air incoming from the east. So the climate in Cracow is characterized by hot summers and cold winters. The mean annual temperature is 8.7 °C and the annual precipitation—750 mm, The prevailing wind directions are western (W) and south-western (SW), and the average wind speed is 1.9 m s^−1^ (Woś 1999).

Growing seasons in the cities differ in length: 210–220 days in Szczecin and over 220 days in Cracow (Woś 1999).

For this study, the volumetric method has been employed using the Hirst spore trap (Hirst 1952) “Lanzoni s.r.l. trademark” with a flow rate of 10 L min^−1^. The traps were installed on the roof tops in the centres of the cities at the same height (20 m) above ground level.

Airborne spores were sampled continuously, 12 months a year during the study period. The Melinex tape used for catching spores was replaced at the same day every week and cut into segments corresponding to 24-h periods. Results (average daily concentration) were expressed as the number of spores m^−3^ air 24 h^−1^.

The daily values of meteorological parameters taken into consideration to assess their effect on the airborne fungal spores were: average air temperature (Av_Temp), maximum air temperature (Max_Temp), minimum air temperature (Min_Temp), relative humidity (RH) and average wind velocity (Av_wind_vel). In Szczecin the meteorological data covering 10 years of the study were provided by the automatic weather station (Vaisala MAWS101 and MAWS201, Finland). The meteorological station was located in the immediate neighbourhood of the aerobiological trap. In Cracow the meteorological data were obtained from the meteorological Web database (http://www.meteo.pl/).

The spore data were analysed to determine the start, the end and the duration (Duration) of the season using the 90 % method. The start of the season (S_Start) was defined as the date when 5 % of the seasonal cumulative spore count was trapped and the end of the season (S_End) as the date when 95 % of the seasonal cumulative spore count was reached (British Aerobiology Federation 1995). Additionally, the following spore season parameters were investigated: average spore concentrations (Av_conc), peak value (Peak_value), peak day (Peak_day) and total number of spores (TNS).

The daily concentrations of spores and the values of meteorological factors formed a 10-year-long time series. Differences between the study sites were assessed with the *U* Mann–Whitney test (1947). Time series trends in the daily concentrations and meteorological parameters were determined using linear regression analysis with the focus on slope coefficients and their significance. Similar analysis was applied for spore season parameters and meteorological parameters averaged for each year. All those calculations were performed in Statistica 10 (StatSoft 2011).

In order to reveal whether changes in the dynamics of spore seasons are driven by meteorological conditions, ordination methods were applied. Ordination primarily endeavours to represent complex, multivariate relationships as correctly as possible in a low-dimensional space (Gauch 1982). The reasons for using that kind of statistical analysis were as follows: (1) the data set included multiple dependent (spore season parameters) and independent variables (meteorological factors); (2) a single multivariate analysis saves time, in contrast to a separate univariate analysis for each dependent variable; (3) ideally and typically, dimensions of the “low-dimensional space” will represent important and interpretable environmental gradients; (4) ordination is a noise reduction technique (Gauch 1982); (5) possibility of determining the relative importance of different gradients (impossible with univariate techniques).

In this study, redundancy among the predictors (meteorological factors) was explored with the variance inflation factor (VIF) in the canonical correspondence analysis (CCA; ter Braak and Prentice 1988) which considered both the environmental parameters and spore season characteristics. VIF analysis is a diagnostic tool used to identify useless constraints. A common rule is that values over 10 indicate redundant constraints (Gross 2003). Using detrended correspondence analysis (DCA) for the dependent variables (spore season characteristics), the gradient of DCA first axis was determined, which indicated the use of either CCA (unimodal responses) or redundancy analysis (RDA, monotonic responses) (Legendre and Legendre 1998; ter Braak 1995) in the final analysis. Significance of consecutive axes was assessed with the ANOVA like permutation test. CCA, DCA and RDA analyses were performed in the R environment (The R Foundation for Statistical Computing 2009) using the CCA (Legendre and Legendre 1998), DECORANA (Hill and Gauch 1980; Oksanen and Minchin 1997), RDA (Legendre and Legendre 1998) and ANOVA.CCA (Legendre et al. 2011) functions of the vegan package.

Prior to ordination analyses, the data were log(*x*)-transformed. The primary reason for such a transformation was that a 1-unit difference in an explanatory variable may potentially has higher importance at its low values than at high level, and then, logarithmic transformation is recommended (Palmer 1993). Moreover, the log transformation unified the levels of dependent parameters and dampened the influence of outliers.

## Results

**Fig. 2:**
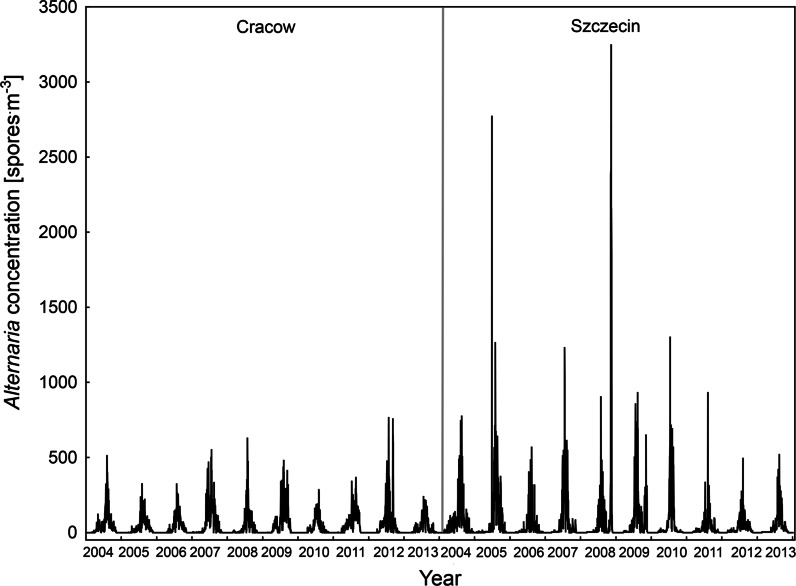
Daily average concentrations of airborne *Alternaria* spores in Cracow and Szczecin in 2004–2013

**Fig. 3:**
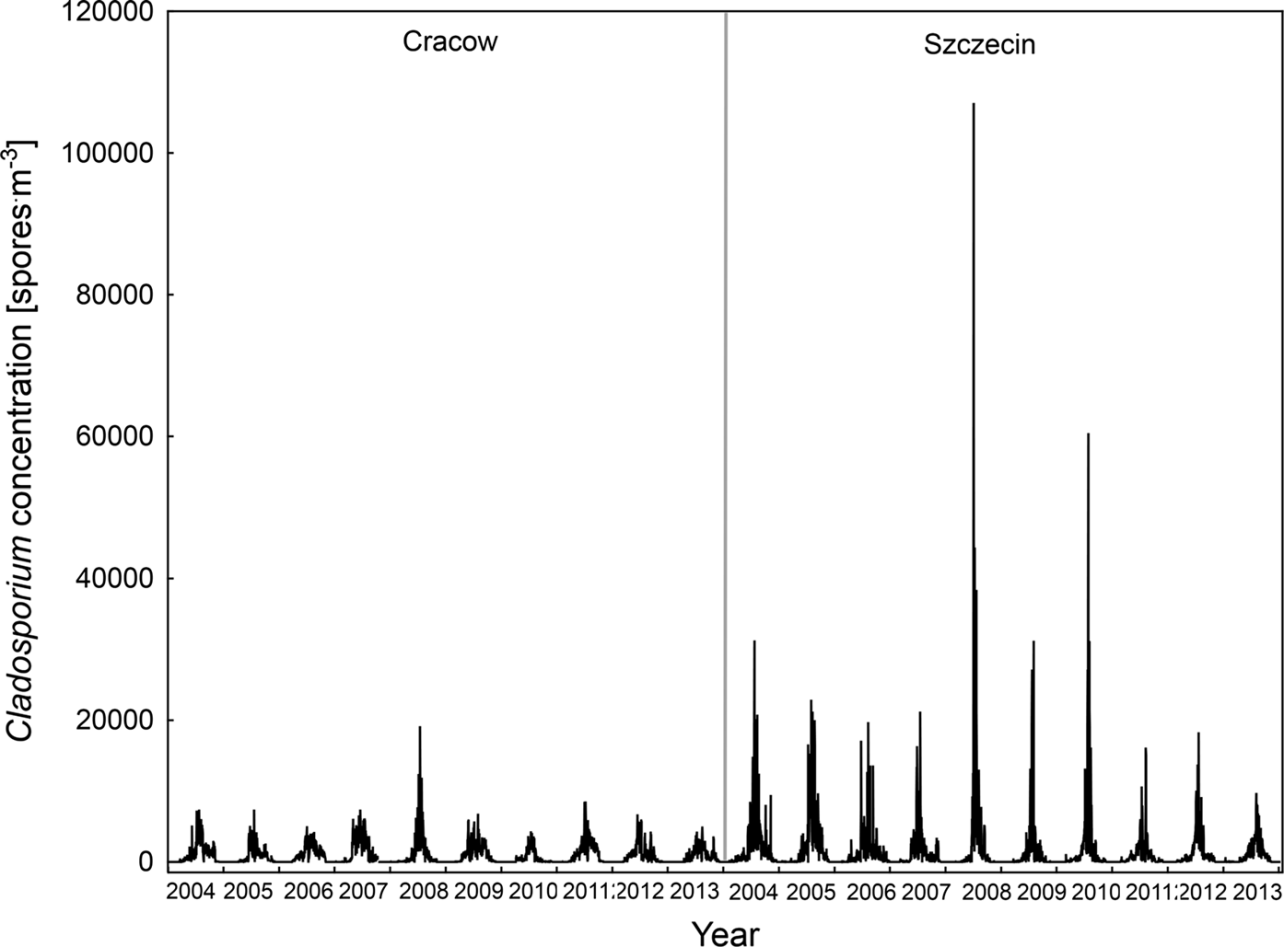
Daily average concentrations of airborne *Cladosporium* spores in Cracow and Szczecin in 2004–2013

Daily a 10-year-long time series of *Alternaria* and *Cladosporium* spore concentrations is presented in Figs. 2 and 3. In general, the abundance of spores was significantly higher in Szczecin comparing to Cracow (Table 1). Due to relatively short spore seasons and the absence of spores for the great part of a year, medians are much lower than the average values. The study sites differed also in meteorological conditions—the average, maximum and minimum temperatures in Cracow were higher but the relative humidity and average wind velocity were significantly lower. Therefore, time series trends were determined with linear regression analysis separately for each site.

**Table 1:** Statistical characteristics of the daily spore concentrations and meteorological parameters recorded in the studied sites in 2004–2013

Variable	Cracow		Szczecin	
	Mean ± SD	Median	Mean ± SD	Median
* *Alternaria* (spores m^−3^)	39.0 ± 72.3	5.0	56.0 ± 161.0	6.0
* *Cladosporium* (spores m^−3^)	882.4 ± 1375.0	205.5	1395.9 ± 3927.5	220.0
* Av_Temp (°C)	9.4 ± 8.6	10.0	8.8 ± 7.8	9.2
* Max_Temp (°C)	14.5 ± 10.1	15.2	13.7 ± 8.9	14.1
* Min_Temp (°C)	5.5 ± 7.8	5.9	4.1 ± 7.0	4.0
* RH (%)	75.7 ± 12.1	77.0	80.4 ± 13.0	82.6
* Av_wind_vel (m s^−1^)	1.6 ± 0.9	1.3	3.2 ± 1.4	3.0

* Statistically significant differences between the sites using *U* Mann–Whitney test (*p* < 0.05)

Linear trends of changes (slope coefficients in linear regression equations) for the daily spore concentrations and daily meteorological conditions in the studied period are presented in Table 2.

**Table 2:** Linear trends of changes in daily spore concentrations and meteorological parameters recorded in the studied sites in 2004–2013

Parameters	*Alternaria* (spores year^−1^)	*Cladosporium* (spores year^−1^)	Av_Temp (°C year^−1^)	Max_Temp (°C year^−1^)	Min_Temp (°C year^−1^)	RH (% year^−1^)	Av_wind_vel (m s^−1^ year^−1^)
Cracow	0.33	−12.48	0.07***	0.11*	0.07***	−0.26**	−0.01
Szczecin	−3.65***	−84.4***	0.03	0.02	0.04	0.01	−0.02**

* Slope coefficients significantly different from zero at *p* < 0.05; ** slope coefficients significantly different from zero at *p* < 0.01; *** slope coefficients significantly different from zero at *p* < 0.001

This analysis revealed the significant decreasing trend in the concentrations of both *Alternaria* and *Cladosporium* spores in Szczecin and the decline in wind velocity. Temperatures increased, however, insignificantly. In turn, the rise in temperatures in Cracow was not accompanied by significant changes in spore abundances.

In the next step, we focused on the parameters describing spore seasons’ characteristics for each year (Table 3) against a background of changes in yearly average meteorological parameters (Table 4).

**Table 3:** Characteristics of spore seasons (average concentration, season start, season end, season duration, peak value, peak day, TNS—total number of spores) recorded in the studied sites in 2004–2013

Site	Taxon	Year	Av_conc (spores m^−3^)	S_Start (dd-mm)	S_End (dd-mm)	Duration (days)	Peak_value (spores m^−3^)	Peak_day (dd-mm)	TNS (spores)
Cracow	*Alternaria*	2004	41.7	02-05	08-10	160	511	05-08	13,791
		2005	30.5	03-06	15-10	135	324	02-08	9841
		2006	28.4	14-06	04-10	113	322	26-07	9338
		2007	56.7	24-05	12-09	112	550	20-07	18,867
		2008	32.9	15-06	15-09	93	627	26-07	10,856
		2009	52.1	04-05	27-09	147	479	04-08	17,215
		2010	27.1	01-05	16-09	139	284	02-08	8958
		2011	44.7	03-05	22-09	143	340	09-07	14,757
		2012	49.9	09-05	25-09	140	764	24-07	16,545
		2013	25.3	06-05	12-10	160	238	19-07	8358
	*Cladosporium*	2004	1000.8	19-05	28-10	163	7075	07-07	330,135
		2005	690.1	14-06	12-10	121	7228	20-07	222,223
		2006	896.2	05-05	14-10	163	4860	02-07	295,345
		2007	1165.4	29-04	30-08	124	5977	17-07	384,817
		2008	992.3	24-05	07-09	107	18,984	15-07	328,229
		2009	982.3	03-05	30-09	151	6640	30-07	323,583
		2010	470.3	28-04	25-08	120	4177	12-07	154,464
		2011	1067.0	06-05	22-09	140	8376	08-07	352,039
		2012	890.6	19-04	25-09	160	5577	09-07	294,302
		2013	663.0	17-05	31-10	168	4814	12-08	218,160
Szczecin	*Alternaria*	2004	62.9	29-04	12-10	167	774	07-08	17,742
		2005	87.9	04-07	04-10	93	1236	30-07	26,140
		2006	32.5	21-06	09-10	111	567	09-08	10,651
		2007	69.3	11-06	20-08	71	1230	16-07	21,511
		2008	90.7	22-06	11-09	51	903	26-07	11,719
		2009	67.6	19-06	15-09	150	930	08-08	19,876
		2010	53.8	11-06	23-08	74	1300	11-07	19,683
		2011	33.2	06-06	04-10	121	930	05-08	11,656
		2012	27.5	04-05	12-10	161	472	02-08	9051
		2013	35.4	12-06	10-10	121	518	12-07	11,549
	*Cladosporium*	2004	1878.3	19-05	28-10	163	31,098	20-07	628,095
		2005	1917.8	14-06	11-10	120	22,737	28-07	675,286
		2006	1276.6	23-05	16-09	178	19,560	06-08	396,063
		2007	1095.9	15-05	06-09	115	21,042	13-07	332,550
		2008	2213.0	21-06	04-09	75	106,896	29-06	738,276
		2009	969.0	30-05	08-09	101	31,054	28-07	348,641
		2010	1803.1	02-06	23-08	83	22,300	30-07	540,216
		2011	822.1	28-04	30-09	185	16,000	05-08	294,439
		2012	1074.7	09-05	28-09	142	18,144	19-07	335,113
		2013	922.2	25-05	02-10	130	9571	31-07	303,596

**Table 4:** Characteristics of yearly average meteorological parameters recorded in the studied sites in 2004–2013

Site	Year	Av_Temp (°C)	Max_Temp (°C)	Min_Temp (°C)	RH (%)	Av_wind_vel (m s^−1^)
Cracow	2004	9.1	13.9	5.4	77.0	1.6
	2005	8.8	13.9	4.9	77.5	1.6
	2006	9.3	14.7	5.2	76.7	1.5
	2007	10.1	15.1	6.2	75.7	1.7
	2008	10.2	15.3	6.3	75.7	1.8
	2009	9.6	14.5	5.7	75.7	1.6
	2010	8.5	13.2	4.8	77.0	1.6
	2011	9.8	15.0	5.7	73.8	1.7
	2012	9.5	14.8	5.4	73.2	1.7
	2013	9.4	14.3	5.7	75.3	1.5
Szczecin	2004	8.6	13.3	3.8	80.6	3.4
	2005	8.8	13.7	4.0	79.8	3.3
	2006	9.3	14.2	4.3	80.1	3.1
	2007	9.6	14.4	4.9	81.9	3.4
	2008	9.4	14.1	4.9	79.8	3.3
	2009	8.7	13.6	3.9	81.5	3.1
	2010	7.3	12.1	2.6	82.2	3.1
	2011	9.4	14.3	4.7	79.1	3.3
	2012	8.7	13.6	3.9	80.9	3.3
	2013	8.8	13.4	4.2	78.5	3.0

The analysis of linear trends of changes in spore seasons and yearly average meteorological parameters revealed only two slope coefficients (Table 5)—*Alternaria* spore seasons in Szczecin tended to appear earlier by around 4 days per year. Humidity in Cracow decreased by slightly above 3 % per year. However, there were a few parameters with only slightly insignificant (*p* level around 0.1) slope coefficients. They indicated the tendency for the earlier start of *Alternaria* and *Cladosporium* spore seasons in Cracow as well as earlier occurrence of the *Alternaria* peak day. For Szczecin, those coefficients were decreasing in spore concentrations and total number of spores.

**Table 5:** Linear trends of changes in spore seasons and yearly average meteorological parameters recorded in the studied sites in 2004–2013

	Parameters	Cracow	Szczecin
*Alternaria*	Av_conc (spores year^−1^)	0.01	−4.46^♦^
	S_Start (days year^−1^)	−2.81^♦^	−4.01*
	S_End (days year^−1^)	−0.87	0.14
	Duration (days year^−1^)	1.94	1.33
	Peak_value (spores year^−1^)	−1.41	−33.94
	Peak_day (days year^−1^)	−1.55^♦^	−1.44
	TNS (spores year^−1^)	10.65	−1016.13^♦^
*Cladosporium*	Av_conc (spores year^−1^)	−17.44	−96.37^♦^
	S_Start (days year^−1^)	−2.62^♦^	−1.78
	S_End (days year^−1^)	−1.19	−0.93
	Duration (days year^−1^)	1.42	−1.08
	Peak_value (spores year^−1^)	−194.36	−1913.71
	Peak_day (days year^−1^)	1.66	0.65
	TNS (spores year^−1^)	−5548.21	−33,796.7^♦^
Meteorological factors	Av_Temp (°C year^−1^)	0.03	−0.04
	Max_Temp (°C year^−1^)	0.03	−0.04
	Min_Temp (°C year^−1^)	0.02	−0.02
	RH (% year^−1^)	−0.34^♦^	−0.08
	Av_wind_vel (m s^−1^ year^−1^)	0.01	−0.02

Slope coefficients significantly different from zero at *p* < 0.05 are marked in *, and slope coefficients at the limit of statistical significance are marked with ^♦^

In order to reveal whether changes in the dynamics of spore seasons are driven by meteorological conditions, the ordination methods were applied. Analyses were performed separately for each spore species. For Szczecin, no significant model was obtained.

**Fig. 4:**
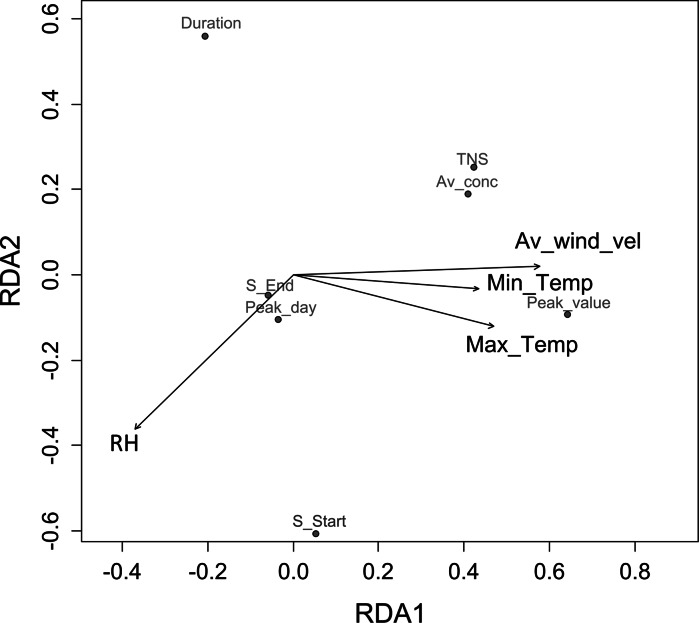
RDA biplot illustrating relations between meteorological variables and *Alternaria* spore season parameters in Cracow in 2004–2013. Significant axis from ANOVA permutations test: RDA1. Variance explained: 42 %

For *Alternaria* in Cracow, the values of VIF coefficients from CCA analysis were <10 for the set of meteorological parameters excluding average air temperature (Max_Temp—4.3, Min_Temp—4.8, RH—1.8, Av_wind_vel—2.5). The next stage of the investigation involved DCA, which was used to determine environmental gradients for subsequent parameters of spore seasons. Values <2 indicated the monotonic responses. Therefore, RDA was performed in the final stage. The ANOVA permutation test confirmed the significance of only RDA1 axis (*p* < 0.05) which explained 42 % of the total variance. Figure 4 presents the obtained RDA biplot.

Air temperatures and wind velocity had positive contribution to RDA1 (Fig. 4, Table 6) while humidity negative. The RDA biplot (Fig. 4) shows that meteorological conditions in a 10-year-long period determined mainly the peak value, the average *Alternaria* concentrations and the total number of spores. Their levels increased with air temperatures and wind velocity and decreased with humidity.

**Table 6:** RDA biplot scores and variance explained by constraining meteorological variables for *Alternaria* and *Cladosporium* spore season parameters in Cracow in 2004–2013

Taxon	Constraining variables	Biplot scores
		RDA1
*Alternaria*	Max_Temp	0.77
	Min_Temp	0.71
	RH	−0.61
	Av_wind_vel	0.94
*Cladosporium*	Max_Temp	0.80
	Min_Temp	0.80
	RH	−0.30
	Av_wind_vel	0.88

**Fig. 5:**
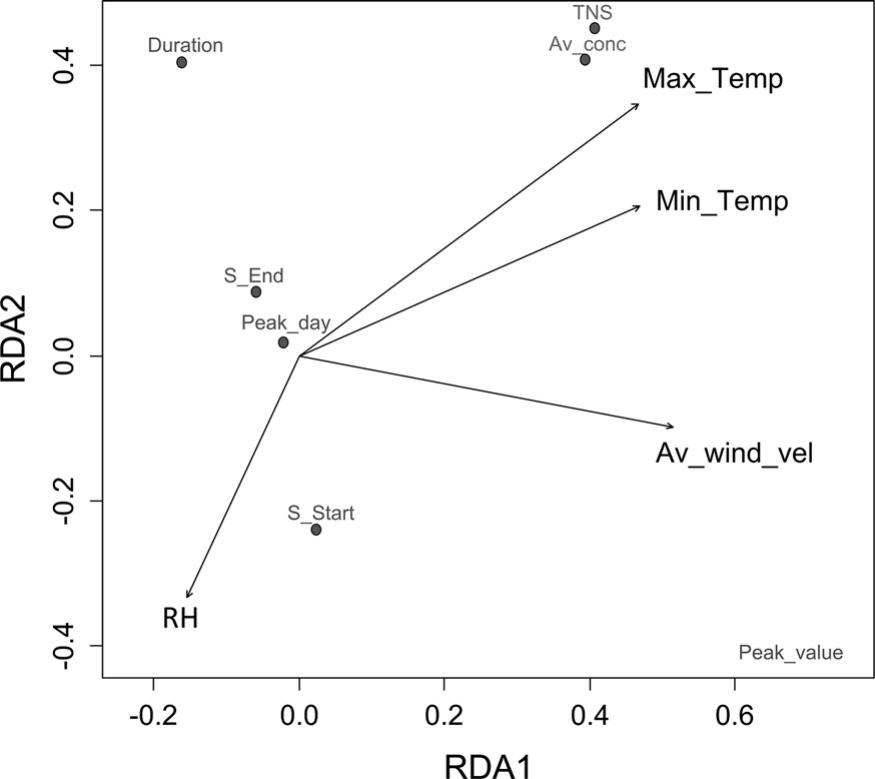
RDA biplot illustrating relations between meteorological variables and *Cladosporium* spore season parameters in Cracow in 2004–2013. Significant axis from ANOVA permutations test: RDA1. Variance explained: 43 %

For *Cladosporium* in Cracow, the values of VIF coefficients from CCA analysis were < 10 for maximum and minimum temperatures, humidity and wind velocity (4.3, 4.8, 1.8 and 2.5, respectively). DCA also indicated monotonic responses of spore season parameters to meteorological parameters (gradient of first DCA axis <2), and then, RDA was performed. The ANOVA permutation test confirmed the significance of only RDA1 axis (*p* < 0.05) which explained 43 % of the total variance. Figure 5 presents the obtained RDA biplot.

RDA1 was mainly connected with air temperatures and wind velocity, and their contribution was positive (Fig. 5, Table 6). The RDA biplot (Fig. 5) shows that meteorological conditions determined mainly the peak value, the average *Cladosporium* concentrations and the total number of spores. Their levels decreased with air temperatures and wind velocity.

## Discussion

Analysis of *Alternaria* and *Cladosporium* daily spores concentration revealed their significantly higher concentrations in Szczecin in comparison with Cracow. It could result from different values of meteorological parameters influencing daily concentrations in Szczecin and could possibly be explained by variability in weather conditions caused by the vicinity of the Baltic Sea.

In Cracow maximum, minimum and average temperatures were higher than in Szczecin, but relative humidity and average wind velocity were significantly lower. The relationship between fungal spore levels and prevailing meteorological factors was observed also by other authors. A strong positive relationship was found between *Alternaria* spore concentrations and mean, minimum and maximum temperature (Angulo-Romero et al. 1999; Sabariego et al. 2000; Burch and Levetin 2002), between concentrations and maximum temperature (Corden and Millington 2001; Mitakakis et al. 2001; Stępalska and Wołek 2005) and between concentrations and mean temperature (Munuera Giner et al. 2001; Troutt and Levetin 2001). Grinn-Gofroń and Rapiejko (2009) suggest that *Alternaria* and *Cladosporium* spore concentrations are mainly influenced by air temperature, but in the spore release a key role play others factors like: changes in relative humidity and wind speed (Sadyś et al. 2014; Fernández-Rodríguez et al. 2015). A strong association between average daily temperature and average daily *Alternaria* and *Cladosporium* spore concentrations was reported also in Zagreb (Peternel et al. 2004). This variability is due to the proximity and abundance of the source of fungal spores and the geobotanical characteristics of studied regions. Some authors indicate the dependence of spore concentrations on the crop production and proximity of grassland areas (Mitakakis et al. 2001; Corden et al. 2003; Pepeljnjak and Segovic 2003). Li and Kendrick (1995) found that concentrations of various fungal spores varied with mean temperature the most, but minimum temperature was the most important in the growing season. The slight correlation between *Cladosporium* daily spore concentration and mean temperature but not significant was noted in Cracow (Southern Poland) by Stępalska and Wołek (2005) and Mitakakis et al. (1997) reported significant, positive correlation with average temperature in Melbourne (Australia).

The sporulation and dispersion of *Alternaria* spores are also closely related to variation in atmospheric conditions. Concentration of *Alternaria* spores in the atmosphere increases when mean temperature rises. Positive, significant correlation with temperature has been reported in other cities around the world (Palmas and Cosentino 1990; Rosas et al. 1990; Fernandez-Gonzales et al. 1993; Hjelmroos 1993; Mitakakis et al. 1997; Munuera Giner et al. 2001; Troutt and Levetin 2001).

Taking the whole our study period into consideration, we observed a decreasing linear trend in concentrations of both taxa spores and in the wind velocity in Szczecin. However, Munuera Giner et al. (2001) found the low wind velocity favouring high spore counts. Pasanen et al. (1991) found that *Cladosporium* spores required an airflow of at least 1.0 m s^−1^ for the spore release. The concentrations of *Cladosporium* spores in forest environments were measured by Kurkela (1997), who noted that the spore release was associated with the reduction in relative humidity and the increase in wind velocity. The highest concentrations occurred at relative humidity of 40–70 % and when wind velocity increased from 0.5 to 1.0 m s^−1^. Lin and Li (2000) reported that fungi concentrations were reduced with increasing wind velocity up to 5 m s^−1^, but at higher wind velocities fungi concentrations increased. Other studies have found no relationship with the wind velocity (Sabariego et al. 2000; Burch and Levetin 2002).

In Cracow we noted a significant increasing trend in temperature, significant decrease in humidity and tendency for the earlier season start and the earlier peak day. The variation in *Alternaria* daily spore concentrations in the atmosphere in relation to meteorological parameters was studied also by Angulo-Romero et al. (1999). Daily concentrations varied positively with temperature and negatively with rainfall, but did not vary with humidity. Daily spore concentrations of *Cladosporium* and *Alternaria* were compared with daily weather data by Katial et al. (1997). *Cladosporium* daily spore concentrations were positively correlated with temperature and relative humidity and negatively with rainfall, while *Alternaria* spore concentrations were found not to be correlated with weather variables. Lyon et al. (1984) reported that once the spores were produced, the release of spores of anamorphic fungi was often greater with higher levels of the minimum wind velocity.

In Szczecin we observed the significantly earlier season start for *Alternaria,* tendency for the decrease in daily spore concentration and total number of spores. However, meteorological conditions did not explain changes in spore season characteristics (insignificant RDA models). The decrease in daily spore concentrations and total number of spores and the earlier season start in Szczecin seems no to be associated with meteorological conditions since they did not change significantly in the study period. In Cracow RDA models indicated that spore season parameters in over 40 % were determined by meteorological conditions, mainly air temperature and wind velocity. If they increase, the peak value, total number of spores and their average concentrations in the season will also increase. The increase in temperature and the decrease in humidity in Cracow had the low impact on daily spore concentration (thus insignificant trends in spore abundance and spore season parameters). However, RDA models indicated the positive relation among the abundance of spores, air temperature and wind velocity. Differences in concentration between two cities are considered to be primarily due to local environmental conditions affecting the local fungi, which stimulate fungal growth.

The effects of temperature, relative humidity and rainfall are important for the source growth and material release. The same meteorological variable may have different effects on different phases of the life cycle, i.e. spore growth, source size, spore emission (Troutt and Levetin 2001). For example, humidity may enhance the spore growth and the increase in the spore size, but it may also reduce emission. The effect of any meteorological variable on spore concentration may differ from year to year because of extremes in other variables.

McCartney (1991) reviewing spore take-off mechanisms and the threshold wind velocity required for spore removal noted that information on the strength of spore attachment and values of threshold wind velocity is not known for the majority of fungi. Dispersal patterns are varied and appear to be different for different species and for the same species at different locations and times.

## Conclusions

1. The study sites (Szczecin, Cracow) differed in higher average, maximum and minimum temperatures and in significantly lower relative humidity and average wind velocity in Cracow.
2. Concentrations of *Alternaria* and *Cladosporium* spores were significantly higher in Szczecin comparing to Cracow.
3. The decreasing trend in spore concentrations was observed in Szczecin.
4. In Szczecin redundancy analysis models did not explain changes in spore season characteristics.
5. In Cracow redundancy analysis models indicated the changes in spore season parameters in over 40 % (caused mainly by air temperature and wind velocity).
